# The acute physiological stress response to driving: A systematic review

**DOI:** 10.1371/journal.pone.0185517

**Published:** 2017-10-16

**Authors:** Michael Antoun, Kate M. Edwards, Joanna Sweeting, Ding Ding

**Affiliations:** 1 Exercise Health and Performance Research group, Faculty of Health Sciences, the University of Sydney, Cumberland, New South Wales, Australia; 2 Charles Perkins Centre, the University of Sydney, Camperdown, New South Wales, Australia; 3 Sydney Medical School, the University of Sydney, Camperdown, New South Wales, Australia; 4 Prevention Research Collaboration, Sydney School of Public Health, the University of Sydney, Camperdown, New South Wales, Australia; Beihang University, CHINA

## Abstract

**Background:**

The experience of driving has been suggested to be detrimental to health. One hypothesis is that each exposure elicits an acute stress response, and that repeated exposures may act as a chronic stressor.

**Objective:**

The aim of this review is to evaluate and synthesise the evidence on whether driving elicits an acute physiological stress response.

**Methods:**

Electronic databases, including CINAHL, PsycINFO and Medline, were searched for original articles written in English from database inception until March 2016. The inclusion criteria of this review included a quantitative examination of an acute physiological stress response to driving, in either on-road or simulated settings, compared to a comparison or control condition. This review followed the Preferred Reporting Items for Systematic Reviews and Meta-Analyses (PRISMA) reporting criteria.

**Results:**

A total of 27,295 abstracts were screened and 28 full-text manuscripts retrieved. Of these, seven articles met the inclusion criteria including four simulator studies and three on-road studies. All suggested a significant change in at least one physiological outcome, but the strongest evidence was for increases in urine catecholamine and cortisol after driving for long hours on-road; results on other outcomes are limited by the small number of studies or inconsistent findings.

**Conclusions:**

Overall, these studies provided moderate evidence to suggest that driving for long hours elicits a stress response over an extended period of time. There is insufficient evidence that driving for a shorter period of time elicits an acute stress response, especially in real, on-road tasks. However, the limited number of studies, small sample sizes, heterogeneity in study objectives, methodologies and physiological outcomes limit conclusions. Future studies could be improved by recruiting a larger sample, utilizing modern stress markers such as heart rate variability, and primarily focusing on the acute physiological stress response to on-road driving.

## Introduction

Cardiovascular disease (CVD) is the leading cause of death globally, accounting for 17.5 million deaths in 2013 [1]. Risk factors for CVD include a genetic predisposition, physiological (e.g., adiposity, hypertension) and lifestyle risk factors (e.g., smoking and physical inactivity), as well as chronic stress. Stress is an ever-present and growing issue in modern societies. According to the Stress and Wellbeing in Australia Survey, conducted by the Australian Psychological Society, as many as 44% of adult participants in 2015 reported increased stress levels over the past 5 years, mainly as a result of personal, financial and health challenges [2].

Lengthy drives to work and other destinations are a common part of modern societies [3], however, it was not until recent years that prolonged driving was proposed as being detrimental to health. To date, only a small number of studies have examined the association between driving time and health outcomes, and most focused on weight-related outcomes. These studies consistently linked driving time with adiposity [4]; for example, Frank and colleagues found a 6% increase in the odds of obesity for each additional hour per day spent in a car [5]. As the relationship between transportation behavior and health is multi-faceted and complex, there has been a recent call for more comprehensive examination of the association between transportation and multiple health outcomes [6]. Based on a large Australian sample, Ding et al. [7] showed that those who drove for more than two hours a day were more likely to have various poor physical and mental health outcomes. Further, a recent UK study found that those who commuted by car and/or public transport had a higher incidence and mortality from CVD as compared with those who commuted actively [8].

Several mechanisms may explain the emerging association between prolonged driving and cardiovascular health. One possible mechanism linking driving and health outcomes is that driving involves prolonged sitting [9], which may compromise cardio-metabolic health [10]. Another proposed mechanism is that the act of driving may evoke an acute stress response similar to that seen in psychological stress responses [11]. Chronic or repeated exposure to acute stressors is associated with increased systemic inflammatory markers and blood pressure [12], which in turn increase the risk of CVD [13].

**Fig 1:**
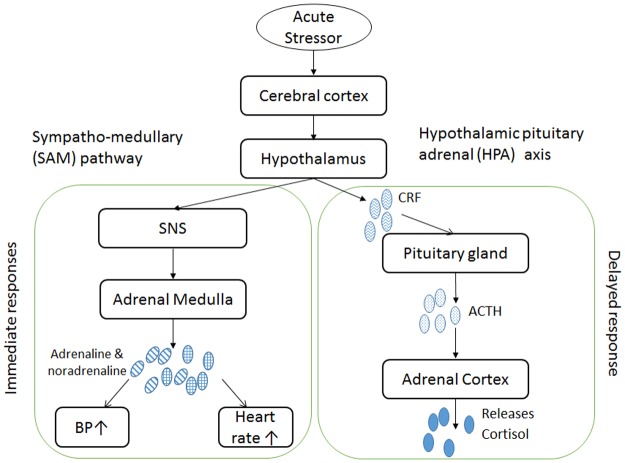
Acute stress response pathways. Abbreviations: ACTH = Adrenocorticotropic hormone; BP = blood pressure; CRF = Corticotropin releasing factor; SNS = sympathetic nervous system; TPR = total peripheral resistance.

To better understand the impact of driving on health, it is important to establish the mechanism of the potential effects. This systematic review was conducted to help elucidate the second proposed mechanism—that driving may impair health through repeated acute stress responses. When examining whether a task evokes an acute stress response, behavioural (e.g. aggression) and physiological responses can be measured. The stress response is controlled by the autonomic nervous system and the hypothalamus-pituitary-adrenal (HPA) axis, involving immediate physiological responses (Fig 1), such as heart rate, heart rate variability, blood pressure, and hormonal responses, such as cortisol release. Changes in these physiological measures are clinically important [14]. For example, a study observing blood pressure changes to acute stressors found that each standard deviation increase in blood pressure responsiveness was associated with a 0.2mm increase in carotid artery thickness [15], which could substantially increase risk for myocardial infarctions [16, 17].

This literature review will examine the current evidence on whether driving, as compared with a non-driving control condition, elicits an acute physiological stress response in adults in experimental or observational studies.

## Methods

### Eligibility criteria

Studies were included in this review if they 1) investigated driving or aspects of driving as the exposure variable, in either an on-road or simulated setting, 2) assessed at least one physiological outcome in adults (18 years of age or older), and 3) included a control or comparison condition. There was no restriction regarding the research design (observational or intervention studies), study location, sample size, date of publication or duration of the driving intervention. Non-empirical manuscript types, including case reports, letters to the editor, editorials, and reviews as well as conference abstracts were excluded.

### Search strategy

A systematic search for full-text research articles written in English was conducted in March 2016 using electronic databases: PsycINFO (1806-present), Medline (1946-present) and CINAHL, with no restriction on year of publication. Driving-related search terms (motor vehicle OR automobile OR commut* OR transport* OR travel OR driving OR driver) and stress response-related search terms (stress OR distress OR cortisol OR heart rate OR blood pressure OR anxiety OR inflammation OR affective OR cardiovascular OR physiological) were combined with an “AND” and searched within the abstract and/or title.

### Study selection

**Fig 2:**
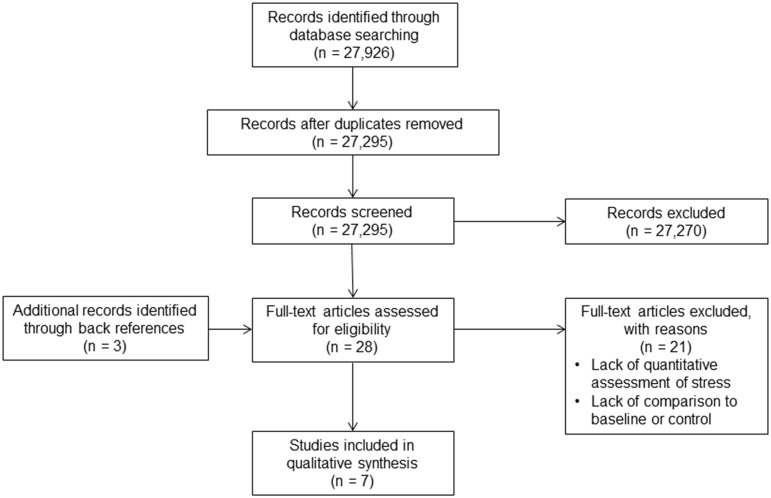
Consort diagram: Study selection.

After removing the duplicates, the title and abstract of each study were assessed by MA and ineligible studies were removed. Backward referencing was then used to identify additional relevant studies. The full texts of remaining studies were assessed independently by two authors (MA, KE), and any disagreement was discussed involving a third author (DD) until consensus was reached among MA, KE and DD. The process of study selection is shown in the CONSORT diagram (Fig 2).

### Data extraction

The following information from each paper was extracted (independently by MA and JS) and summarized in Table 1: authors, publication year, setting (where not specified, setting was assumed to be the first author’s affiliation), objectives of the study (e.g. investigating the physiological stress response to driving), study design (experimental vs. observational), participant characteristics (age, gender, and health status), sample size, protocol, physiological outcomes and results. We also extracted additional details on the measurement instruments, such as electrocardiography (ECG) for heart rate, and the timing of measurement. In order to demonstrate the overall patterns of associations, we further distilled study findings in Table 2, using symbols (↑ indicating a significant increase, ↓ for a significant decrease and a ↔ for no statistically significant change). Funding sources for each study were extracted. All data extracted were presented separately for simulator and on-road studies.

**Table 1:** Summary of included studies that assess the acute physiological changes in response to either on-road or simulated driving.

First author, year (Setting)	Study design	Objective	Participants	Protocol	Measures, timing and techniques	Results
**Ashton, 1972 (UK)** [23]	Randomized crossover	Measure motor-perceptual performance and physiological response of human subjects simultaneously under various conditions	n = 15 non-smokers (60% M), age range: 19-26yrs, mean age: 20.8yrs	Simulation of 3 levels of driving difficulty (from Level 1[control] to Level 3), allocated in randomized order, 20min each.	Continuous measures of heart rate (ECG) and blood pressure (semi-automatic blood pressure cuff)	1) ↑ in heart rate from Level 1 to 3. Significant difference between control (Level 1) and Level 3 (mean 81.23 beats/min v. 84.35 beats/min, p<0.05); 2) **↔** in blood pressure.
**Seeman, 1995 (USA)** [20]	Pre-post	Investigate gender differences in patterns of HPA response to a driving simulation challenge	n = 26 healthy participants (46% M), age range: 70–79 yrs, mean age: 72.3 yrs	Two ‘drive-along’ simulations; one covering usual driving situations (18min), the other involving rapid response to avoid an accident (10min)	Blood sampling at -10, 0, +20 minutes (between films) and +45min (after 2nd film): 1) ACTH (two site immunoradiometric assay); 2) Cortisol (radioimmunoassay)	1) ↑ mean ACTH (p = 0.04). Mean max response = +5.03pmol/l; 2)↑ mean cortisol (p<0.001). Mean max response = +19.12mmol/l
**Yamaguchi, 2007 (Japan)** [21]	Pre-post	Evaluate the usefulness of sAMY as an indicator of the acute psychological effects of driving	n = 20 (100%F) with no oral disease, age range 20–23 yrs, mean age: 21.4 yrs	Study 1–5 minutes of baseline followed by 21 minutes of normal simulated driving. Study 2 –as above with addition of navigation device operation	3 minute measures throughout driving task (total = 7) of Salivary amylase (hand-held monitor with disposable saliva test strips)	1) Study 1: ↑ sAMY of 45.2% (baseline mean = 15.7 kU/l; driving mean = 22.8kU/l); 2) Study 2: ↑ sAMY of 30.6% (baseline mean = 15.7kU/l; driving mean = 20.5kU/l)
**Yamakoshi, 2009 (Japan)** [22]	Pre-post	Evaluate the use of salivary CgA, as a mental stress marker, in response to the stressful situation created by simulated monotonous driving	n = 25 healthy participants (gender not stated), age range not stated, mean age: 26.8 yrs	10 minutes baseline followed by 120 minutes (max.) of stress inducing simulated driving	Continuous measures of: 1) Blood pressure, total peripheral resistance and normalised pulse volume by finger photo-plethysmograph; 2) Heart rate and cardiac output by ECG; 3) 10 minute saliva measures of CgA by ELISA	1) ↑ mean blood pressure; 2) (24%) and total peripheral resistance (22%); 3) **↔** mean heart rate or cardiac output; **4) ↓** mean CgA (1%) and mean normalised pulse volume (35%); 5) (% estimated from Figure 3 in [22])
**Aronsson, 1998 (Sweden)** [18]	Observational	Investigate the psychophysiological stress reactions in female and male urban bus drivers	n = 20 urban bus drivers (50%M) age range not stated, mean age: 30.1 yrs	Eight hour driving shift and an eight hour control day watching educational TV programs (1–2 wks post driving session)	hourly measures of: 1) Adrenaline and noradrenaline (urine, by photofluorimetric method); 2) Cortisol (urine, by radioimmunoassay); 3) Blood pressure (Cardy 8 Mini electronic metre)	Driving v rest (pmol/min/kg): 1) ↑ mean adrenaline by approx. 100% (women: 0.601 v 0.306; men: 0.885 v. 0.513); 2) ↑ mean noradrenaline by approx. 50% (women: 2.991 v 2.331; men: 3.011 v 2.222); 3) ↑mean cortisol by approx. 50% (women: 4.211 v 3.681; men 4.131 v 3.942); **4) ↔** in blood pressure
**Bellet, 1969 (USA)** [19]	Randomized crossover	Investigate the effect of driving on catecholamine and 11-OHCS (cortisol) urinary excretion	GROUP A n = 17 normal subjects (gender not stated), age range:19–25 yrs, mean age not stated GROUP B n = 19 subjects with coronary artery disease (gender not stated), age range: 38–72 yrs, mean age not stated	120 minutes of driving compared to 120 minutes of rest	2 hourly measures of: 11-OHCS (cortisol) (urine, by fluorometric method) and Catecholamines (urine, by trihydroxyindole fluorometric method)	Rest v driving (μg): 1) ↑ mean catecholamines (Grp A: 2.86 v 4.35; Grp B: 4.48 v 7.15); 2) ↑ mean 11-OHCS (Grp A: 21.1 v 30.0; Grp B: 20.8 v 35.5)
**Sluiter, 1998 The Netherlands** [24]	Pre-post	Evaluate work stress and corresponding recovery by means of urinary excreted adrenaline, noradrenaline, and cortisol in long distance coach drivers	n = 10 (100%M) coach drivers, age range not stated, mean age: 47 yrs	Examined urinary excretion rates of adrenaline, noradrenaline and cortisol during three consecutive driving days compared to two consecutive days off.	3–4 hourly measures (n = 7) of: 1) Adrenaline and noradrenaline (urine, by high performance liquid chromatography with fluorescence detection); 2) Cortisol (urine, by high performance liquid chromatography on a C_18_ column with UV detection)	Comparing first work day and baseline (ng.min^-1^): 1) ↑ mean adrenaline (9.52 v 6.73); 2) ↑ mean noradrenaline (49.50 v 41.98); 3) ↑ mean cortisol (20.97 v 15.78); 4) (means calculated from numbers reported in paper)

Abbreviations: 11-OHCS– 11-hydroxycorticosteroid; ACTH–adrenocorticotrophic hormone; CgA–Chromogranin- A; ECG–electrocardiogram; ELISA–Enzyme linked immunosorbent assay; HPA–hypothalamic-pituitary-adrenal axis; M–Male (M); sAMY–salivary amylase; UK–United Kingdom; USA–United States Of America

**Table 2:** Summary of the physiological outcomes measured in the included studies.

	Hypothalamic-pituitary-adrenal axis		Sympathetic nervous system						
	Cortisol	ACTH	Catecholamines	Heart rate	Normalised pulse volume	Blood pressure	Total peripheral resistance	Salivary amylase	CgA
**Simulated driving studies**									
**Ashton (1972)**				**↑**		**↔**			
**Seeman (1995)**	**↑**(plasma)	**↑**(plasma)							
**Yamaguchi (2007)**								**↑**(saliva)	
**Yamakoshi (2009)**				**↔**	**↓**	**↑**	**↑**		**↓**(saliva)
**On-road driving studies**									
**Aronsson (1998)**	**↑**(urine)		**↑**(urine)			**↔**			
**Bellet (1969)**	**↑**(urine)		**↑**(urine)						
**Sluiter (1998)**	**↑**(urine)		**↑**(urine)						

↑ indicates a significant increase, **↓** indicates a significant decrease and ↔ indicates no significant change in the physiological variable comparing driving, either on-road or simulated, to a baseline or control.

Catecholamine has been used as an umbrella term which includes adrenaline and noradrenaline.

Abbreviations: ACTH—adrenocorticotrophic hormone, CgA–Chromogranin A

Due to heterogeneity in study design and outcome measures, a meta-analysis was neither possible nor appropriate, thus a systematic review detailing key findings of individual studies was conducted. Our review followed the PRIMSA (Preferred Reporting Items for Systematic Reviews and Meta-Analyses) reporting criteria.

## Results

### Study selection

The initial search generated 27,926 potentially relevant articles, as shown in Fig 2. After removing 631 duplicates, 27,295 papers remained. Of those, 27,182 were removed based on reading the title, 88 were removed based on reading the abstracts and an additional three papers were identified from backward referencing. Twenty-one papers were excluded after reading the full text, leaving a total of seven articles for inclusion in this review. Primary reasons for exclusion based on full text included lack of quantitative assessment of stress or comparison to a baseline or control. The majority of studies (5 of 7) were funded by government/research grants [18–22]. One study was sponsored by a Tobacco Research Council [23] and one did not specify funding sources [24].

### Study characteristics

#### Study design and setting

The methodological details of the seven studies that met the inclusion criteria are presented in Table 1. Of those, four were simulated driving studies and three were on-road studies. The simulator studies included one randomized crossover study, and three pre-post studies. The on-road studies included one randomized crossover trial, one pre-post study and one observational study. The randomized crossover studies included a driving session and a control/comparison session, of which the order was randomly assigned. In pre-post studies, the control session always preceded the driving session. The observational study did not manipulate driving conditions and only compared outcome measures on working days with non-working days among bus drivers. The studies were set in Japan (n = 2), the United States of America (n = 2), and Europe (n = 3).

#### Participants

In total, these studies included 162 participants, with sample sizes ranging from 10 to 36, with a mean of 23 participants per study. The age of the participants where included (four studies) ranged from 19 to 79 years, with a mean age of 36.4 years (six studies). Within the five studies reporting gender, 41 (45%) participants were male and 50 (55%) participants were female.

Five studies recruited participants from the general population and two studies recruited bus/coach drivers. Of the two studies among professional drivers, one required participants to be familiar with the driving route with a minimum of one year’s driving experience [24], and the other study restricted professional driving experience to between two and five years [18]. Six studies included only ‘healthy’ participants, with this definition ranging from having no oral disease, having been medically screened, to an absence of cardiovascular disease.

#### Objectives

Five studies examined the physiological response to normal driving [18, 19, 21, 23, 24]. Of these studies, one further tested the effect of gender [18] and another tested the effect of coronary artery disease on the stress response to driving [19]. Two studies aimed to determine the physiological stress response to a designated stressful driving task [20, 22].

### Simulated driving studies

The study by Ashton et al. [23] involved three levels of difficulty. Level 1 (the control) required participants to respond to light signals alone, Level 2 required participants to respond to light signals which were reinforced by movement of the car in the simulated film, and Level 3 involved participants responding to light signals, which at times conflicted with the movement of the car. Each session was 20 minutes in duration. The light signals corresponded to a certain action that the participant was required to complete, i.e. a green light signal indicated the participant should switch on their indicator, whilst a red light indicated that the brake pedal must be pressed. Subjects in the study by Seeman et al. [20] were seated in a simulated console, which resembled the driving seat of a car, with one of two films projected onto the wall in front of the subjects. The participants were required to ‘drive along’ with both films, with each sitting preceded by a two-hour “rest” or “recovery” period. The first film, titled “good driving strategies”, was 18 minutes long and involved steering, turning and changing lanes. The second film, titled “evasive action control”, was ten minutes long and involved breaking and rapid turning to avoid a crash. Yamaguchi et al. [21] incorporated two sub-studies, with Study 1 involving five minutes of baseline rest followed by 21 minutes of simulated driving and Study 2 including an additional task, which required input of a series of numbers into a touch panel, designed to replicate the use of a navigation device. The participants within the study by Yamakoshi et al. [22] were required to sit in a driving seat and rest for ten minutes. The participants were then instructed to drive the simulator monotonously for 120 minutes at a set speed, as if they were actually driving. The driving session ended prematurely if the participant left the specified lane.

### On-road studies

The study by Aronsson et al. [18] required bus drivers to drive predetermined “moderately stressful” routes for their usual eight-hour shift. This session was then compared with eight hours spent viewing educational television programs. Participants within the Bellet et al. [19] study were required to drive two hours and this was compared with two hours of seated rest. The bus drivers in the Sluiter et al. [24] study were all given an identical route to drive, which consisted of three days of driving, and this was compared with two days of rest.

### Outcomes

Cortisol was measured in four studies, of which three measured urinary cortisol [18, 19, 24] and one plasma [20]; catecholamine excretion was measured in three studies all of which were urinary measures [18, 19, 24]; blood pressure was measured in three [18, 22, 23] and heart rate in two [22, 23]. Less commonly used (in one study only) markers of stress included normalised pulse volume, total peripheral resistance, salivary Chromogranin-A [22], as well as adrenocorticotrophic hormone (ACTH) [20] and salivary amylase, a marker of the sympathetic nervous system response [21]. The results of the physiological outcomes from each study are summarised in Table 2.

### Syntheses of results

#### Simulated driving studies

The simulator studies evaluated various physiological measures comparing the simulated driving task to the baseline or control. Two studies measured blood pressure, with one study finding increased blood pressure during driving when using a continuous recording [22] and the other finding no change when using intermittent recordings [23]. Two studies measured heart rate continuously, and both showed no significant change [22, 23]. One study showed significant increases in average adrenocorticotrophic hormone (ACTH) and cortisol excretion (urine), measured at intervals of -10min, 0min, 20min (between driving simulations) and 45min after the second driving simulation [20]. One study showed a significant increase in average salivary amylase whilst driving compared to the control, measured at three-minute intervals [21]. The last study recorded continuous total peripheral resistance (increased) and normalised pulse volume (decreased), and found a significant decrease in average chromogranin-A (CgA), measured at 10-minute intervals [22].

#### On-road driving studies

All three studies measured both catecholamines (including adrenaline and noradrenaline) and cortisol, and consistently found an increase in both measures. One study used a single measurement taken after two hours of driving [19], one study averaged two samples taken two hours apart [18] and one study reported a mean daily measure consisting of multiple samples [24]. One study also measured blood pressure during the last four hours of an eight hour driving shift, and showed no significant change in average blood pressure over two recordings, two hours apart [18].

## Discussion

This is the first review to our knowledge that summarizes the literature on the acute physiological stress response to driving. The current research evidence on this topic is limited, as demonstrated by the small number of studies identified. Based on these studies, we conclude that there is moderate evidence suggesting that driving for long hours elicits a stress response over an extended period of time. There is, however, insufficient evidence that driving for a shorter period of time elicits an acute stress response, especially in real, on-road tasks.

This review found very limited evidence for on-road driving, where only long-duration exposures were examined; investigations using driving simulation tasks offer more varied durations of exposure. There was moderate support for the activation of the HPA axis by driving with urinary excretion of cortisol consistently elevated compared with non-driving control conditions in all three on-road studies using extended driving exposures. Evidence of HPA activation was also found in one simulation task study, which reported elevated plasma cortisol measured acutely after the driving task. It is known that only about 1% of free blood cortisol is excreted in the urine, limiting the validity of urinary measures [25]. There is no evidence currently for increase in “free cortisol” (the biologically active component, measured most often in saliva) stimulated by driving.

Evidence for the activation of the sympathetic-adrenal-medullary (SAM) axis is similarly consistent in long duration on-road studies using urinary excretion of catecholamines. However, indices of outcomes measuring acute changes (seconds or minutes, rather than much longer duration changes measured by urinary excretion) in SAM mediated outcomes are less consistent. Continuous measures of heart rate during simulated driving found increases in one study and no change in another, similarly, blood pressure was found to be increased only in one of the two studies that measured its response in simulated driving. Other markers of sympathetic nervous system activation measured by single studies (including total peripheral resistance [22], salivary amylase [21], chromogranin-A (CgA) [22] and normalized pulse volume [22]) showed similarly mixed findings.

The stress response to driving, if any, has been suggested to be related to certain aspects, components or situations of driving. Several studies were designed to explore this research question. For example, in the study by Ashton et al [23], the addition of conflicting light signals resulted in an increase in heart rate, which was not observed in the normal simulated driving task, suggesting that the cognitive demand associated with the conflicting light signals may have led to the increase in heart rate. Yamaguchi et al. [21] required participants to use a navigation device in addition to the simulated driving in the second part of the study and found that although an increase in salivary amylase was observed, it was lower compared with the simulated driving task alone. A decline in mental concentration is expected to be associated with inactivation of sympathetic nervous activity, and the authors suggest that operating distracting devices, such as navigators and mobile phones, may reduce drivers’ capacity to focus on driving. These findings may suggest an association between stress responses to driving impedance, as certain situations, such as traffic congestion, parking difficulty, and negative interactions with other drivers, are likely to increase cognitive demand and the stress perceived [7].

Acute stress response to driving may also differ by the attributes of drivers, such as health status and sociodemographic characteristics. The study by Bellet et al. [19] observed participants with an established diagnosis of coronary artery disease, and showed that they had a greater physiological response to the driving task, possibly due to the over reactivity of the sympathetic nervous system observed in patients with coronary artery disease. The study by Seeman et al. [20] aimed to examine stress response by gender, and found that women experienced more prolonged elevation in cortisol. Previous studies indicated that human salivary cortisol responses might differ by sex [26]. As there is currently little research on differential stress response to driving by driver characteristics or driving situations, future studies should fill this gap by investigating how driving affects individuals differently. In particular, it is important to determine the role of driving regularity. In two of the three on-road driving studies, participants were professional drivers (bus/coach drivers) and their stress response to driving may differ from non-professional drivers, due to their repeated exposures over a long period.

### Limitations

It is important to acknowledge that the conclusions from the current systematic review are based on a small number of studies with heterogeneous study designs and outcomes measured. Several limitations apply, but more to the empirical studies than the review process. Specifically, simulated driving studies may lack validity when compared with on-road studies, due to the fact that they do not have the same degree of risk as on-road driving [27]. Simulators may also result in different stress responses from on-road driving due to “simulator sickness” [28], a common syndrome that involves nausea, dizziness and vomiting whilst performing simulated tasks. Despite better validity and resemblance to everyday driving situations, findings from the on-road studies should still be interpreted with caution. Two studies were conducted in long distance bus drivers, suggesting that the results may not be generalizable to everyday car driving [18, 24]. It has been shown that dealing with potentially dangerous customers, night shifts and potential health complications associated with bus driving could lead to stress [29]. All three on-road studies measured urinary cortisol and catecholamines, which may not capture an acute stress response to driving, as previously discussed. Despite the use of a randomized crossover trial by Bellet et al. [19], the study is dated. The current driving environments, including traffic congestion [30] and distractions including navigators [21], are distinctively different from those at the time of the study (1969), limiting the generalisability of the findings to the driving experience nowadays. On the other hand, given that it is reasonable to assume that the driving situation today may involve more “driving impedance” than the 1960s, one may conclude that the significant stress response found in this study could be underestimated.

### Recommendations for future studies

Given the problem of car dependency and the continuous increase in car use in many societies, as observed in Australia [31, 32], it is critical to improve the current understanding of the health implications of driving. Given the complex nature of driving with health and wellbeing [6], studies should continue to investigate various health outcomes of driving and their related mechanisms, including stress response to driving. Both epidemiological and physiological studies are indispensable in this inquiry. Here, we provide several suggestions for future physiological studies that aim to quantify stress response to driving exposure as a key step towards understanding the mechanisms through which driving may impair cardiovascular health.

One major gap in the literature is the lack of state-of-the-art outcome measures whilst performing on-road driving, such as salivary cortisol, heart rate and heart rate variability (HRV). The use of salivary sampling for cortisol will be a major improvement from the current studies as it better captures “free cortisol” as compared with plasma and urinary measures, and it measures immediate physiological response to driving as an acute stressor. In addition, HRV is a commonly used biomarker that measures the flexibility and balance of the autonomic nervous system in dealing with stressors [33], such as mental stress tasks and work stress [34]. Second, as synthesised results from the current review suggest a difference between short bouts of driving and long hours of driving, it raises the question regarding whether a “threshold effect” exists in terms of driving-induced stress, for example, how many hours of uninterrupted driving would make driving a significant stressor? Third, characteristics such as age, genetic predisposition and various lifestyle behaviors can affect the automatic nervous system and reduce HRV permanently, which is in turn associated with a range of cardiovascular diseases [35]. Future studies may benefit from recruiting larger and more diverse samples to examine whether HRV responses to driving differ by the individual characteristics listed above. Moreover, to further elucidate the potential link between driving and CVD outcomes, additional studies are needed to examine intermediate outcomes between acute stress response and CVD, such as maladaptation and dysregulation induced by repeated exposures to the stressor.

## Conclusions

The literature regarding the physiological response to driving is limited. Based on a small number of studies, there is moderate evidence suggesting that driving over an extended period could elicit SAM and HPA activation but there is limited evidence supporting driving as an acute stressor. However, this conclusion is limited by a small number of studies, small sample sizes and the heterogeneity in the objectives, methodologies and physiological outcomes. Given the ubiquitous and habitual nature of driving and the strong evidence suggesting that repeated exposure to acute stressors may lead to adverse health outcomes, particularly CVD, more research is needed to understand the stress related to driving and car dependency and its long-term implications on health.

## Supporting information

S1 Table PRISMA checklist. (DOC)
